# Mechanistic modeling for agroecological adaptation: genetic potential, uncertainty, and *in silico* design

**DOI:** 10.3389/fbinf.2026.1886248

**Published:** 2026-07-07

**Authors:** Edgar S. Correa, Francisco C. Calderon

**Affiliations:** School of Engineering, Pontificia Universidad Javeriana, Bogota, Colombia

**Keywords:** agroecological transition, bioinformatics, crop adaptation, genetic parameters, *in silico* design, mechanistic modeling, systems biology, uncertainty

## Abstract

Biological adaptation is increasingly studied through data-rich descriptions of environments, organisms, and their interactions. In crop systems, this challenge is especially concrete: genetic potential, physiological regulation, soil–water dynamics, climate variability, and management decisions jointly determine performance across heterogeneous landscapes. Mechanistic crop models provide a way to organize this information because their parameters, equations, and assumptions make biological interpretation possible. Yet current practice still emphasizes calibration and prediction more strongly than the explicit evaluation of mechanistic adequacy under independently characterized environments, quantified uncertainty, and competing computational descriptions. This Perspective argues that mechanistic models are most useful for agroecological transitions when treated as explicit and revisable hypotheses about biological dynamics. Using rainfed rice under drought as a methodological application, it discusses how environmental domains can be defined before crop response is modeled, how uncertainty and structural adequacy can be examined, how artificial intelligence can complement rather than replace mechanistic reasoning, and how genetically grounded parameter spaces can support *in silico* exploration of adaptation options. It therefore proposes a transparent evidence-to-design framing in which environmental characterization, mechanistic representation, model evaluation, and *in silico* adaptation design are treated as connected components of a single analytical workflow. This framing aligns crop modeling with broader questions in bioinformatics, systems modeling, and ecosystem dynamics: how exchanges of genetic, environmental, physiological, and computational information can be represented in ways that remain transparent enough for scientific scrutiny and useful enough for design.

## 1 Introduction

Agricultural adaptation is a biological, environmental, and computational problem. Crops express genetic potential, understood here as the biologically meaningful range of genotype-related capacities represented through model parameters, through physiological processes that unfold under changing water, energy, nutrient, and management conditions. The resulting performance is not the direct effect of one factor, but the outcome of interactions among genotype, environment, and management across space and time (Hammer et al., 2014; Lobell et al., 2011; Jägermeyr et al., 2021; Hultgren et al., 2025). This makes crop systems a useful scale for examining a broader scientific question: how biological dynamics can be observed, represented, evaluated, and used for design without losing interpretability.

Mechanistic crop models have become essential tools for formalizing this problem. They translate knowledge of phenology, growth, resource capture, biomass accumulation, partitioning, and yield formation into explicit computational structures (Muller and Martre, 2019; Peng et al., 2020; Hoogenboom, 2025). This places crop modeling within a broader systems-biology tradition in which computational models are used to connect mechanism, measurement, and system-level behavior (Kitano, 2002). Despite important advances in predictive crop modelling, integrative frameworks that explicitly connect independent environmental characterization, uncertainty analysis, AI-assisted model evaluation, and biologically interpretable design remain limited, especially when models are expected to support mechanistically accountable interpretation rather than prediction alone.

Their contribution is often judged by predictive performance, and prediction remains indispensable for decision support. Yet their deeper scientific value lies in a more demanding property: they expose assumptions about how biological and environmental processes are connected. Because those assumptions are explicit, they can be evaluated, challenged, and revised. This Perspective advances the central argument that the unresolved need is not simply for better prediction, but for a more explicit analytical framing that links environmental structure, mechanistic representation, uncertainty-aware evaluation, and design-oriented interpretation within a transparent evidence-to-design workflow. This distinction is particularly relevant for a Research Topic concerned with genetic and communicative exchanges shaping ecosystem dynamics. At the crop scale, information exchange is not limited to gene flow or organismal communication. It also includes the transmission of environmental signals through soil, water, climate, and associated biotic communities; the expression of genetically influenced traits through physiological parameters; the interaction between management and biological response; and the use of computational models to translate these relationships into testable hypotheses. This interpretation is consistent with broader evidence that plant performance emerges from genetic, biochemical, physical, metabolic, and environmental interactions across plant-associated systems (Trivedi et al., 2020). A crop model can therefore be read not only as a prediction engine, but as a structured representation of how information moves across genetic, physiological, environmental, and decision-making layers.

Rainfed rice under drought is used here as a bounded methodological case rather than as the exclusive object of the argument. It allows a wider problem to be examined: how independently characterized environments, uncertainty-aware model evaluation, AI-based discrepancy analysis, and genotype-oriented *in silico* exploration can be integrated into a transparent evidence-to-design workflow. The argument is grounded in the current crop-modeling and systems-biology literature and illustrated with recent peer-reviewed applications (Hammer et al., 2014; Challinor et al., 2018; Reichstein et al., 2019; Peng et al., 2020; Correa et al., 2025; Correa, 2026b; Correa, 2026a). Its purpose is broader than the rice case itself: to define when mechanistic models can support agroecological adaptation with scientific rigor, biological interpretation, and design-oriented transparency. More broadly, the manuscript asks under what conditions mechanistic models remain useful as revisable scientific structures: open to interrogation, refinement, and reinterpretation as environmental characterization improves, uncertainty becomes more explicit, and new forms of biological evidence become available.

**FIGURE 1:**
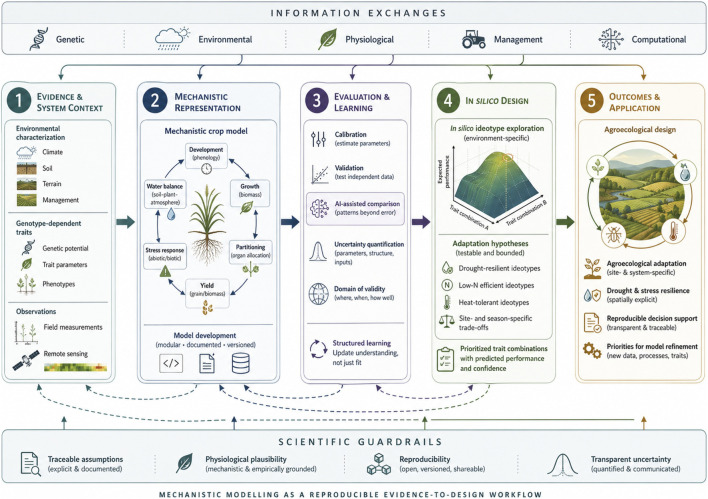
Mechanistic modelling as a reproducible evidence-to-design workflow for agroecological adaptation. Genetic, environmental, physiological, management, and computational information are organized across five linked stages: evidence and system context; mechanistic representation; evaluation and learning; *in silico* design; and outcomes for agroecological application. The lower band highlights scientific guardrails that sustain interpretability and rigor: traceable assumptions, physiological plausibility, reproducibility, and transparent uncertainty. The workflow positions mechanistic crop modelling as an explicit framework for integrating heterogeneous evidence, evaluating model adequacy, and generating bounded adaptation hypotheses.

Figure 1 synthesizes the central argument as a reproducible evidence-to-design workflow in which environmental characterization, mechanistic representation, model evaluation, and *in silico* design are treated as connected components of a single analytical framework.

## 2 Mechanistic models as explicit hypotheses

A mechanistic model differs from a purely statistical predictor because it encodes a proposed structure of causation. Parameters are not only numerical coefficients; many represent biological traits, management choices, or environmental constraints. In crop models, genotype-specific coefficients may describe phenological development, partitioning, potential grain number, growth duration, or sensitivity to temperature and photoperiod (Hoogenboom, 2025; Muller and Martre, 2019). These parameters are not genes themselves, but they provide a quantitative interface through which genetic potential can be represented, compared, and explored under environmental variation, consistent with multiscale crop-modeling efforts that seek to connect biological mechanisms with field- and regional-scale adaptation (Peng et al., 2020). The same formal requirement appears across biological domains whenever mechanistic models are used to represent regulated growth, signaling, metabolic dynamics, or morphogenetic change through explicit process structure rather than statistical association alone.

This interface is valuable precisely because it is incomplete. A useful mechanistic model does not need to represent every biological detail. It must instead be explicit enough for its domain of validity to be examined. The model should make clear which processes are represented, which are simplified, which parameters are calibrated, which data support those parameters, and which outputs are being interpreted. In this manuscript, mechanistic accountability refers to the requirement that a model user be able to state which process is represented, which assumptions support that representation, which evidence constrains it, and under which conditions its interpretation remains valid. In this sense, mechanistic modeling is not a closed description of reality. It is a disciplined way of stating a biological hypothesis in computational form. This is one reason why mechanistic modeling remains relevant across scales and domains of biological inquiry: the same demand for explicit representation reappears whenever measured variables must be linked to proposed processes in an interpretable way.

Mechanistic models can also become opaque when treated as compact computational objects. Inputs, parameters, state variables, empirical closures, and outputs may then be analyzed together as if they had the same evidential status. This obscures the difference between a variable available in the software and a mechanism biologically represented by the model. A variable becomes mechanistically interpretable only when it can be linked to a represented process, an equation or algorithmic rule, constraining measurements, explicit assumptions, and a domain of validity. Expert use therefore cannot be reduced to running the software, calibrating parameters, or examining all outputs after simulation. It requires mechanistic accountability in practice: the capacity to state which process is being invoked, which model component represents it, which assumptions make the inference reproducible, and where the formulation should not be treated as biological evidence.

Such a view changes how model performance is interpreted. A high predictive score can be useful, but it is not by itself sufficient evidence that the modeled mechanisms are adequate. Conversely, imperfect prediction does not make a model uninformative if the discrepancy reveals where representation, data, or assumptions require refinement. The most productive use of mechanistic models is therefore not to defend a formulation, but to learn from the conditions under which it succeeds, fails, or behaves unexpectedly.

## 3 Environmental structure before biological response

Rigorous interpretation begins before simulation. If environments are grouped through observed yield or through stress classes generated by the same crop model being evaluated, the environmental domain becomes partly dependent on the response it is meant to explain. This can obscure the distinction between environmental structure and biological performance. A more stable approach is to define the target environments through independent biophysical information, such as soil, terrain, climate, and water availability (Hammer et al., 2014).

This principle guided the rainfed rice application. Environments were first characterized using soil and climate attributes before crop response was interpreted (Correa et al., 2025). Terrain-driven hydrological variability was then resolved through a Runoff Potential Index, allowing drought exposure to be considered in relation to landscape structure rather than as a spatially uniform condition (Correa, 2026b). More broadly, independent characterization may also be strengthened through richer crop- and landscape-level observation regimes, including multispectral segmentation, feature extraction, plant-scale reconstruction, and image-based multispectral characterization (Colorado et al., 2020; Jimenez-Sierra et al., 2021; Correa et al., 2020; Correa et al., 2022; Correa et al., 2024). This step matters beyond the specific case. It separates the physical context in which adaptation occurs from the biological response that the model seeks to explain. It also creates a stronger basis for reproducibility, because model behavior can be evaluated against environmental domains defined independently of model outputs.

For agroecological transitions, this distinction is practical as well as epistemological. Adaptation options are credible only when the environmental conditions to which they apply are explicit. Recommendations about sowing dates, water management, cultivar choice, or trait combinations must be linked to the environments where they are expected to operate. Without that link, model outputs risk becoming generic prescriptions detached from the heterogeneity that determines their usefulness.

## 4 Uncertainty, adequacy, and reproducibility

Mechanistic modeling becomes scientifically stronger when uncertainty is treated as part of the analysis rather than as a final caveat. Parameter uncertainty, input-data quality, spatial resolution, calibration strategy, model structure, and domain of application all affect interpretation. These sources of uncertainty do not have the same meaning. Some can be reduced by better data or calibration; others indicate that relevant biological processes are absent, simplified, or active outside the model’s intended range (Asseng et al., 2015; Wallach et al., 2018).

The rice application illustrates this distinction. Calibration and parameter optimization established a reproducible mechanistic baseline, but performance reached a plateau under the studied rainfed conditions (Correa et al., 2025). This result is important because it suggests that additional parameter tuning alone may not resolve all limitations. The point is not that the model is deficient in a general sense. The point is more precise: under particular environmental and physiological conditions, the adequacy of the formulation must be evaluated against independent evidence.

Reproducibility is central to that evaluation. A mechanistic workflow should report input data, assumptions, parameter ranges, calibration procedures, uncertainty treatment, environmental domains, and criteria for interpreting outputs. This is especially important when model results inform adaptation, breeding, or policy. Reproducibility does not guarantee correctness, but it allows other researchers to understand how conclusions were reached, compare alternative formulations, and identify where improvement is most needed.

## 5 Artificial intelligence as a test of mechanistic representation

Artificial intelligence can improve prediction, but its most valuable role in this context may be diagnostic. In Earth and biological systems, data-driven methods are increasingly valuable when they help recover spatio-temporal structure and support process understanding rather than simply replacing mechanistic reasoning (Reichstein et al., 2019). When data-driven methods outperform a mechanistic model, the difference should not automatically be interpreted as evidence for replacement. It can instead indicate where the mechanistic formulation is not capturing relevant structure in the observed system.

In the rainfed rice application, machine learning improved predictions of yield and biomass relative to the mechanistic baseline (Correa et al., 2025). The scientific interest of this result was not the gain in fit alone. It became meaningful because the improved estimates were compared with physiological expectations for aerobic or non-flooded rice systems. This comparison helped distinguish numerical improvement from biological plausibility. In that sense, AI served as a complementary lens for evaluating model adequacy.

This role is well aligned with bioinformatics and systems modeling. As environmental sensing, phenotyping, imaging, and data integration become more detailed, AI can help identify patterns that are not yet fully represented mechanistically. Imaging-centered systems biology has already shown how richer observation can make dynamic biological processes more traceable across scales (Megason and Fraser, 2007). The challenge is to use those patterns without abandoning causal interpretation. A productive workflow should therefore ask whether data-driven corrections point to missing processes, altered parameter domains, scale mismatches, or context-specific interactions that can guide model refinement.

Comparable tensions between predictive performance, interpretability, and mechanistic understanding are increasingly visible across computational biology, including domains concerned with gene regulation, signaling, and other dynamic biological processes. In genomics, explainable artificial intelligence has been proposed as a way to extract biologically meaningful insight from deep learning systems rather than treating prediction as an endpoint in itself (Novakovsky et al., 2023). At the same time, the broader methodological critique of black-box models has made clear that *post hoc* explanation is not always a sufficient substitute for interpretable modelling when scientific or high-stakes interpretation matters (Rudin, 2019). This concern is especially relevant in biology, where models often function not as direct mirrors of reality but as explicit and revisable descriptions of how we currently organize causal understanding (Gunawardena, 2014). From this perspective, the value of AI is greatest when it complements mechanistic modelling by revealing missing structure, constraining plausible dynamics, or guiding refinement, rather than replacing mechanistic description with opaque prediction alone.

## 6 Genetic potential and *in silico* adaptation design

Mechanistic models are also useful because they make design questions tractable. When genotype-related parameters are represented explicitly, it becomes possible to explore virtual combinations of traits across defined environmental domains. The result of such exploration is not a new cultivar, but a modeled optimum: a virtual ideotype, understood here as a modeled optimum in parameterized trait space predicted to perform well under a defined environmental domain and model structure, rather than as a direct breeding product. Such ideotypes should be understood as interpretable hypotheses about adaptive strategies.

The genotype-oriented *in silico* phenotype-optimization analysis combined sensitivity analysis with genetic-algorithm exploration across virtual cultivars to identify environment-specific ideotypes under contrasting rainfed conditions (Correa, 2026a). In relation to earlier crop-design and multiscale modeling work (Hammer et al., 2014; Peng et al., 2020), the analysis is used here for a narrower but important purpose: to show how environment-specific optimization can remain interpretable when sensitivity analysis, virtual ideotype exploration, and similarity to field-characterized germplasm are treated as connected steps. The contribution was not simply the identification of high-performing virtual ideotypes. It showed how a mechanistic parameter space can connect environmental heterogeneity, physiological interpretation, and genotype-oriented design.

This is where the link with genetic information becomes especially clear. Crop-model parameters can act as a bridge between observed performance and genetically influenced traits, even when the model does not resolve molecular mechanisms. They allow researchers to ask which trait configurations are plausible, which environmental domains they serve, and which gaps remain between available germplasm and modeled optima. For agroecological transitions, this matters because adaptation cannot depend only on external inputs or short-term management. It also requires biological strategies matched to heterogeneous environments and evaluated through transparent methods. At the same time, virtual ideotype approaches have important limitations. First, the optima they identify are conditional on the model structure, parameterization, assumptions, and environmental domain used in the analysis; they should therefore be interpreted as conditional model-based hypotheses rather than general biological conclusions. Second, even when the parameter space is biologically grounded and restricted to realistic values, a virtual ideotype does not necessarily correspond to an existing cultivar. Its practical value may therefore lie in identifying plausible target combinations for pre-breeding or longer-term trait assembly, rather than in pointing directly to material already available for deployment. Third, virtual ideotypes may inherit structural biases from the underlying model when relevant processes are absent, simplified, or weakly constrained by available evidence. Finally, different objective functions, imposed constraints, or environmental scenarios may lead to different optima, so the result should be interpreted as a conditional adaptation hypothesis rather than as a final prescription. Their value lies less in prescribing an ideal cultivar than in making adaptation hypotheses explicit, comparable, and open to revision under improved data, alternative model structures, and stronger links between parameterized traits and genetic evidence.

## 7 Recommendations for rigorous application

The following principles summarize the practical implications of the argument. They are not proposed as a fixed protocol, but as conditions that can improve interpretability, reproducibility, and usefulness across mechanistic modeling studies (Box 1). They reflect broader state-of-the-art concerns in crop modeling and systems modeling: defining target environments before response interpretation, making assumptions auditable, comparing model structures, and using data-driven methods to interrogate biological representation rather than obscure it (Hammer et al., 2014; Challinor et al., 2018; Reichstein et al., 2019; Peng et al., 2020; Correa et al., 2025; Correa, 2026b; Correa, 2026a).

Box 1 **Conditions for using mechanistic models in agroecological design.**

1. Define the environmental domain independently. Characterize soils, climate, terrain, and water-related constraints before interpreting crop response or model-generated stress classes.
2. Make mechanisms, not only variables, explicit. Distinguish inputs, state variables, parameters, empirical closures, and outputs; report which processes are represented or simplified, and which parameters are calibrated, fixed, borrowed, weakly constrained, or inactive in the target domain.
3. Separate prediction from adequacy. Evaluate fit together with physiological coherence, literature consistency, and performance across environmental domains.
4. Use AI diagnostically. Treat data-driven improvements as opportunities to identify missing structure, scale mismatches, or context-specific responses.
5. Preserve reproducibility. Document data sources, preprocessing, parameter ranges, calibration routines, uncertainty analyses, and code availability whenever possible.
6. Connect ideotypes to biological interpretation. Treat virtual ideotypes—modeled optima in parameterized trait space—as hypotheses about adaptive strategies, not as final prescriptions.

These principles are relevant to agronomy, biology, engineering, bioinformatics, and decision-oriented research because they clarify what must be true before a model output becomes useful evidence. They also help communicate the value of mechanistic modeling to funders and institutions: the goal is not only to produce simulations, but to build transparent analytical systems that can support adaptation decisions, reveal uncertainty, and guide future data collection.

## 8 Discussion

Mechanistic modeling is most valuable when it remains open to revision. This position recognizes the achievements of existing crop models while also identifying the conditions under which they can become more informative. Models such as DSSAT and CERES-Rice have provided a durable basis for simulating development, growth, and management responses across many environments (Hoogenboom, 2025; Muller and Martre, 2019). Their continued relevance depends on using them with the same rigor that made them scientifically useful: explicit assumptions, careful parameterization, independent evaluation, and transparent reporting.

The perspective developed here is therefore constructive. It does not ask mechanistic models to become complete representations of biological reality. It asks that they be used in ways that make their strengths and limits visible. When environmental domains are independently defined, when uncertainty is analyzed rather than hidden, when AI is used to interrogate representation, and when genotype-related parameters are explored as biological hypotheses, mechanistic models can support more than prediction. They can become tools for explanation, comparison, and adaptation-oriented design. This also clarifies why the rainfed rice case is not only an application, but a bounded entry point into a broader problem: how biological models can remain interpretable while moving from evidence to design.

This view also connects crop-scale modeling with broader ecosystem questions. Genetic potential, physiological regulation, environmental signals, management actions, and computational inference are different forms of information that interact through time. Representing those interactions requires more than larger datasets. It requires models whose assumptions can be examined and whose outputs can be interpreted in relation to biological mechanisms and environmental context.

For agroecological transitions, this is a practical requirement. Decisions about resilient crops, resource use, and sustainable production need tools that are not only accurate under selected conditions, but also transparent about when and why they work. Mechanistic models can help meet that need when they are embedded in reproducible workflows that integrate observation, uncertainty, biological interpretation, and design. The crop scale offers a concrete application, but the underlying lesson is broader: biological modeling gains scientific and social value when it makes complex dynamics more explicit, more testable, and more useful for responsible action. In this sense, the Perspective does not close the question; it sharpens the next one. Future research should extend this framework in at least four directions. First, stronger links are needed between parameterized traits and independent genetic or physiological evidence, so that genotype-related model structure can be interpreted with greater biological precision. Second, richer observation regimes—including phenotyping, imaging, and other multiscale measurements—should be used not only to improve calibration, but to refine which processes are represented, how their domain of validity is defined, and where important mechanisms remain absent. Third, hybrid workflows combining mechanistic models with data-driven components should be developed in ways that preserve interpretability and support refinement rather than replacement of causal structure. Finally, the broader promise of this perspective lies in its transferability across levels of biological organization, including contexts in which growth, resource allocation, regulation, and morphodynamic change must be represented as measurable, revisable, and designable biological processes. The frontier is not only to make models more predictive, but to make biological dynamics increasingly measurable, interpretable, and designable across scales.

## Data Availability

The original contributions presented in the study are included in the article/supplementary material, further inquiries can be directed to the corresponding author.
